# NTBC Treatment of the Pyomelanogenic *Pseudomonas aeruginosa* Clinical Isolate PA1111 Inhibits Pigment Production and Increases Sensitivity to Oxidative Stress

**DOI:** 10.1007/s00284-014-0593-9

**Published:** 2014-05-07

**Authors:** Laura M. Ketelboeter, Vishwakanth Y. Potharla, Sonia L. Bardy

**Affiliations:** Biological Sciences, University of Wisconsin–Milwaukee, Milwaukee, Wisconsin USA

## Abstract

Pyomelanin is a brown/black extracellular pigment with antioxidant and iron acquisition properties that is produced by a number of different bacteria. Production of pyomelanin in *Pseudomonas aeruginosa* contributes to increased resistance to oxidative stress and persistence in chronic infections. We demonstrate that pyomelanin production can be inhibited by 2-[2-nitro-4-(trifluoromethyl) benzoyl]-1,3-cyclohexanedione (NTBC). This treatment increases sensitivity of pyomelanogenic *P. aeruginosa* strains to oxidative stress, without altering the growth rate or resistance to aminoglycosides. As such, NTBC has potential to function as an anti-virulence factor in treating pyomelanogenic bacterial infections.

**Electronic supplementary material:**

The online version of this article (doi:10.1007/s00284-014-0593-9) contains supplementary material, which is available to authorized users.

## Introduction

*Pseudomonas aeruginosa* is an environmental bacterium that is capable of causing both acute and chronic infections in compromised patients. This organism is extremely adaptable, has a high level of intrinsic antibiotic resistance, a wide range of virulence factors, and the ability to form biofilms (reviewed in [1] ). Antibiotics are an essential part of treating *P. aeruginosa* infections, but the inherent resistance combined with emerging resistance due to selective pressure limits the therapeutic options available. As a new strategy to combat infectious disease, the specific inhibition of virulence factors has been proposed as an alternate treatment mechanism [2]. By attenuating bacterial virulence without targeting essential bacterial pathways, it may be possible to aid in the clearing of infections while minimizing selective pressures that perpetuate resistance.

Pyomelanin, a dark brown/black pigment, is a potential target for anti-virulence compounds. Pyomelanin production has been reported in *P. aeruginosa* isolates from urinary tract infections and chronically infected Cystic Fibrosis (CF) patients [3, 4]. Pyomelanin is one of the many forms of melanin that is produced by a wide variety of organisms. Production of pyomelanin is reported to provide a survival advantage, scavenge free radicals, bind various drugs, give resistance to light and reactive oxygen species, and is involved in iron reduction and acquisition, and extracellular electron transfer [4–9]. A number of environmental and pathogenic bacteria have been reported to produce this pigment [3, 8, 10–14]. In *Shewanella oneidensis* and *S. algae*, pyomelanin plays a role in biogeochemical cycling of metals, as pigment production enhances hydrous ferric oxide reduction and electron transfer [15–17]. In *Legionella pneumophila*, pigment production may contribute to pathogenesis as pyomelanin mediates ferric reduction from ferritin and transferrin [8]. Non-pyomelanogenic strains of *Burkholderia cepacia* are more sensitive to externally generated oxidative stress and show reduced survival in phagocytic cells [11]. In *P. aeruginosa*, pyomelanin production results in increased persistence and virulence in mouse infection models [3].

**Fig. 1:**
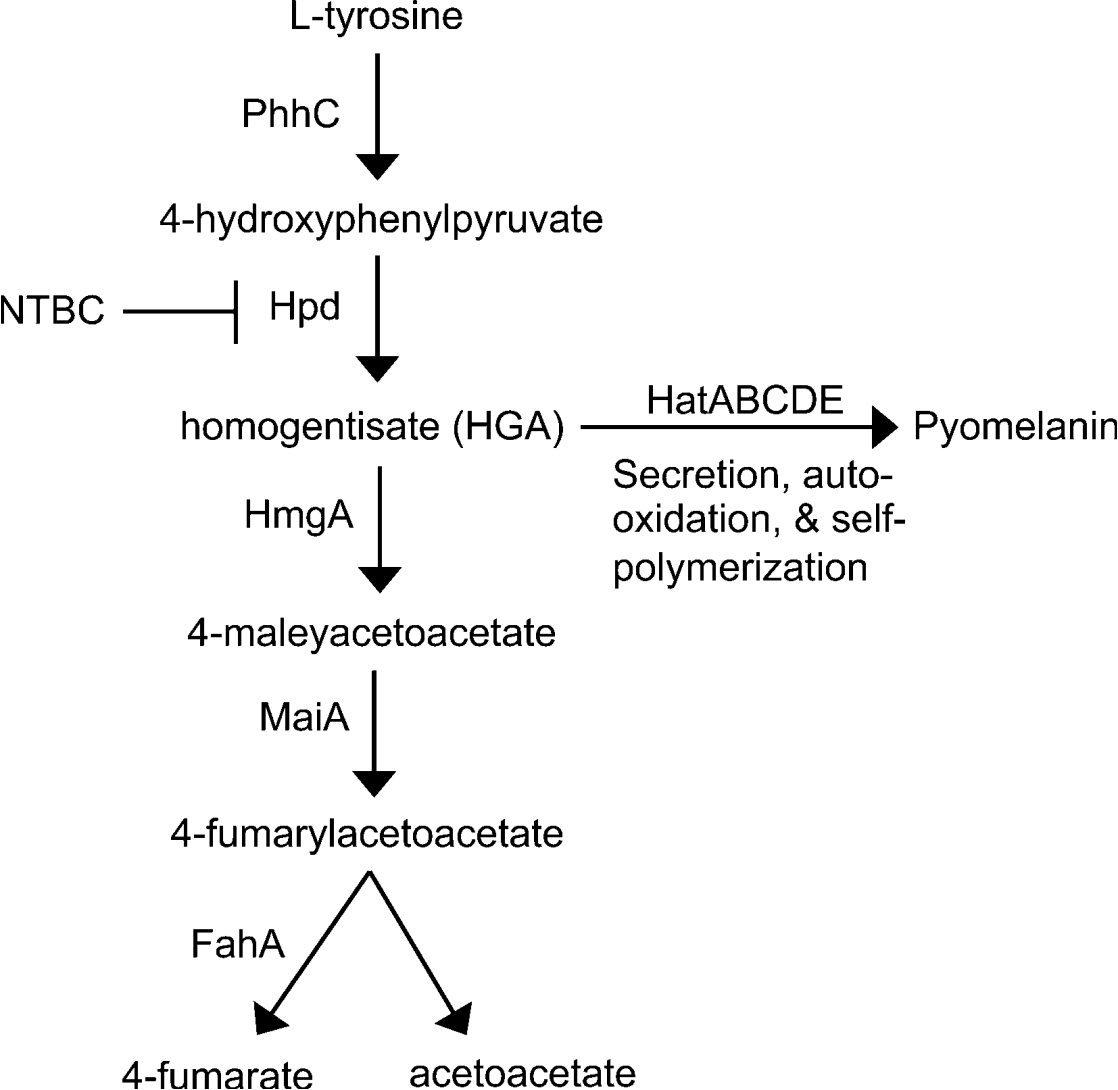
Tyrosine catabolism pathway of *Pseudomonas aeruginosa*. Inactivation of HmgA results in the secretion of HGA, which auto-oxidizes and self-polymerizes to form pyomelanin. NTBC inhibits HGA production and pyomelanin formation through interactions with Hpd

Pyomelanin is a negatively charged extracellular pigment of high molecular weight, derived from the tyrosine catabolism pathway [6, 18, 19]. 4-Hydroxyphenylpyruvate is converted to homogentisate (HGA) by 4-hydroxyphenylpyruvate dioxygenase (Hpd) (Fig. 1). HGA is then converted to 4-maleylacetoacetate by homogentisate 1,2-dioxygenase (HmgA). A loss of HmgA activity leads to the accumulation of HGA, which is secreted via the ABC transporter HatABCDE. Defects in either the ATP-binding cassette or the permease components of this transporter result in reduced pyomelanin production [4]. Once secreted from the cell, HGA auto-oxidizes and self-polymerizes to form pyomelanin. Both point mutations in *hmgA* and chromosomal deletions have been reported in clinical *P. aeruginosa* isolates producing pyomelanin [3, 10].

Hpd activity is essential for the synthesis of HGA, and ostensibly irreversible binding with 2-[2-nitro-4-(trifluoromethyl)benzoyl]-1,3-cyclohexanedione (NTBC) inhibits Hpd activity of *Streptomyces avermitilis* in vitro [20]. Although it was originally developed as an herbicide, NTBC is a FDA-approved treatment for type I tyrosinemia [21]. Type I tyrosinemia is the result of a defect in the tyrosine catabolism pathway, which causes the accumulation of toxic metabolites such as fumarylacetoacetate, leading to cirrhosis and cancer of the liver [22]. Binding of NTBC to Hpd prevents the accumulation of toxic metabolites and disease progression [21]. We report here on NTBC treatment of pyomelanogenic strains of *P. aeruginosa*, the resulting reduction in pyomelanin production, and the corresponding increase in sensitivity to oxidative stress.

## Materials and Methods

### Bacterial Strains and Growth Conditions

Laboratory strains of *P. aeruginosa* PAO1 (obtained from Carrie Harwood, University of Washington), transposon mutants *hpd::tn* (PW2577) and *hmgA::tn* (PW4489)) and the clinical isolate PA1111 were grown at 37 °C in LB supplemented with tetracycline (60 μg/ml) and gentamycin (50 μg/ml) as appropriate. The transposon mutants were obtained from the University of Washington transposon mutant collection [23]. *Escherichia coli* DH5α (NEB) was used as a host for recombinant plasmids, and was grown in LB with gentamycin (10 μg/ml) as appropriate.

### Chemicals

NTBC (2-(2-nitro-4-trifluoromethylbenzoyl)-1,3-cyclohexanedione), H_2_O_2_, and tobramycin were purchased from Sigma-Aldrich. Gentamycin and kanamycin were purchased from Gold Bio and Fisher Scientific, respectively.

### Growth Curves

Overnight cultures were grown in LB + 300 μM NTBC or LB with the corresponding amount of DMSO. The overnight cultures were diluted to OD_600_ 0.05 in LB + 300 μM NTBC or LB + DMSO, and the optical density was measured every hour. Each sample was pelleted and resuspended in LB prior to the optical density reading to ensure that the results were not influenced by the presence of pyomelanin.

### Oxidative Stress Assay

Overnight cultures were grown with NTBC (300 μM) or with a corresponding volume of DMSO as a control. Optical densities (OD_600_) were measured using washed cells, and cultures were diluted to equivalent OD_600_ values (~2.5). Tenfold serial dilutions were made in PBS containing either 300 μM NTBC or DMSO as appropriate. 5 μL of each serial dilution was spotted onto LB plates containing the indicated concentration of H_2_O_2_. Laboratory strains were incubated for 24 h and PA1111 was incubated for 45 h at 37 °C.

### Determination of MICs

Minimal inhibitory concentrations (MICs) were determined by twofold serial microtiter broth dilution [24], using an inoculum of 2.75 × 10^5^ CFU/ml. Inoculum concentration was determined using washed cells to ensure that pyomelanin production did not affect OD_600_ readings. NTBC was included in the appropriate wells at a final concentration of 300 μM. MICs were recorded as the lowest concentration of antibiotic inhibiting growth following 24 h of incubation at 37 °C.

## Results and Discussion

### Pyomelanin Production by a Clinical Isolate of *P. aeruginosa*

PA1111, a pyomelanogenic clinical isolate from an acute infection, was obtained from Dara Frank (Medical College of Wisconsin). This strain lacked type III secretion proteins but was cytotoxic in a tissue culture assay [25]. To determine the cause of pyomelanin production in this isolate, HmgA (PA2009) from PAO1 was expressed from pJN105 [26]. Following induction with 0.05 % and 0.1 % arabinose, pyomelanin production was eliminated in *hmgA::tn* and PA1111, respectively (Online resource 1a). *P. aeruginosa* *hmgA::tn* functions as a positive control for pyomelanin production as *hmgA* is interrupted with the IS*phoA/hah* transposon [23]. *P. aeruginosa* PAO1 and *hpd::tn* (isogenic to *hmgA::tn*) were included as negative controls; neither strain produces pyomelanin.

Since increased amounts of arabinose were required to eliminate pyomelanin production in PA1111 relative to *hmgA::tn* (compare 0.05–0.1 %), we assayed *hpd* transcript levels through RT-PCR (Online resource 1b). Quantification of the relative levels revealed that in both PAO1 and PA1111 *hpd* transcript was approximately 10 % more abundant than in *hmgA::tn*. It is unlikely that this subtle increase in *hpd* transcript levels is responsible for the residual pyomelanin production in PA1111 at low levels of induction (0.05 % arabinose). This suggests that the clinical isolate may have altered translation or post-translational modification resulting in increased expression or activity of Hpd.

The ability to abolish pyomelanin production in PA1111 through expression of wild-type HmgA suggested that either a chromosomal deletion or inactivation of the *hmgA* gene occurred, both of which have been reported in clinical isolates of *P. aeruginosa* [3, 10]. A third reported cause of pyomelanin production is imbalanced enzyme expression within the l-tyrosine catabolism pathway. In *Vibrio cholerae* (ATCC 14035), homogentisate dioxygenase and the downstream enzymes are expressed at lower levels than hydroxyphenylpyruvate dioxygenase, leading to an accumulation of HGA and pyomelanin production [27]. To determine the genetic cause of pyomelanin production in PA1111, we attempted to PCR amplify and sequence *hmgA*. Despite repeated attempts, we were unable to amplify *hmgA* via colony PCR. To verify these results, Southern hybridization was performed with DIG-labeled *hmgA* as a probe. No hybridization was detected between the *hmgA* probe and the PA1111 genome (data not shown). This, combined with our ability to complement the pyomelanin phenotype via induction of *hmgA* expression, suggests that a chromosomal deletion has occurred.

### NTBC Inhibits Pyomelanin Production in *Pseudomonas aeruginosa* Without Disrupting Growth

**Fig. 2:**
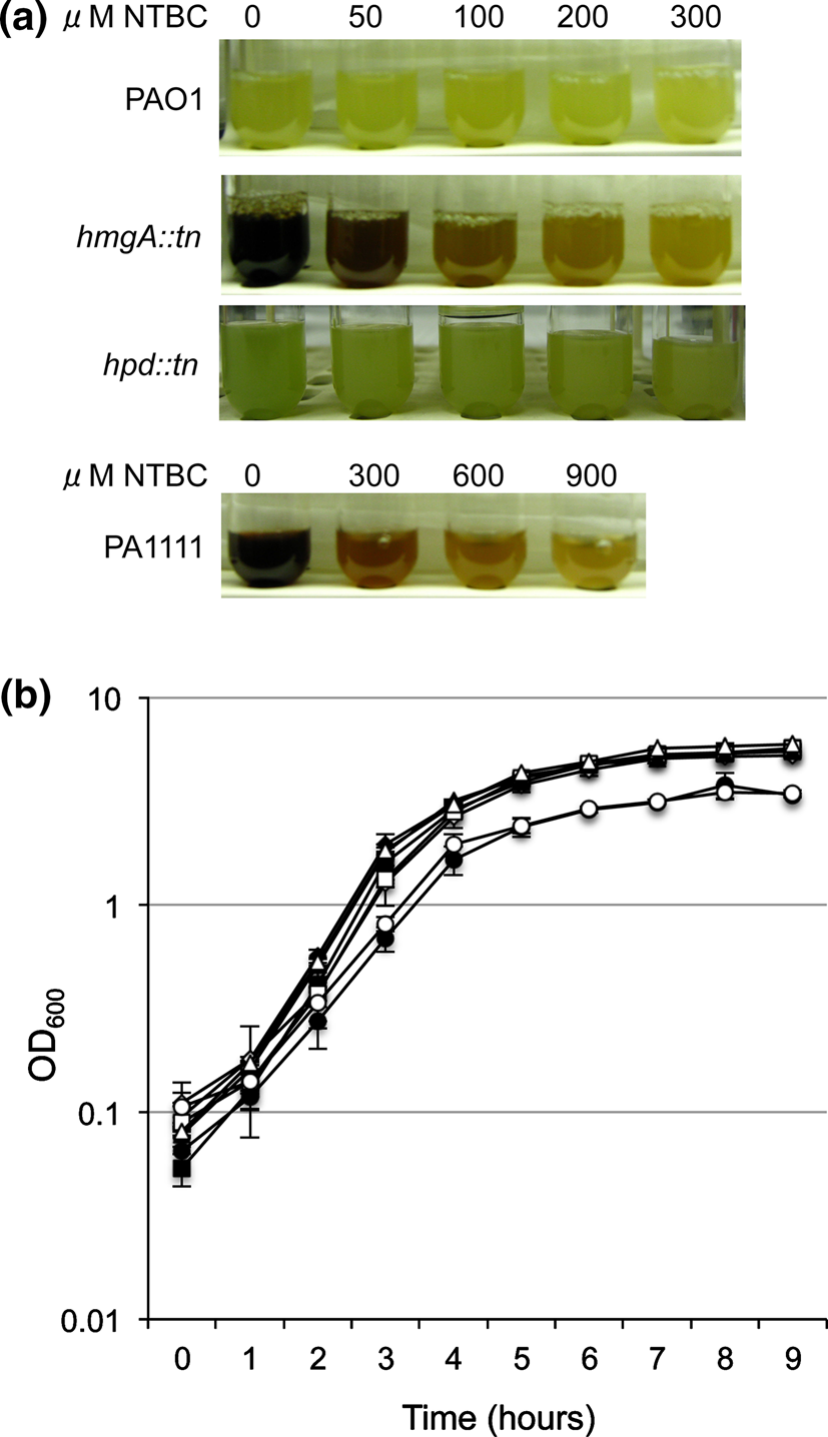
NTBC treatment inhibits pyomelanin production without affecting growth. **a** Pyomelanin production by *P. aeruginosa* with and without NTBC treatment. Laboratory and clinical strains were grown overnight with the indicated concentrations of NTBC. **b** Growth curves of laboratory and clinical strains of *P. aeruginosa* with and without 300 mM NTBC treatment. Strains grown without NTBC are indicated with closed symbols, while those grown with NTBC are indicated with open symbols. Wild-type PAO1 (diamonds), *hpd::tn* (triangles), *hmgA::tn* (squares), PA1111 (circles). The growth curves are compiled from three independent experiments, with error bars indicating standard error of the mean

NTBC is known to bind Hpd (4-hydroxyphenylpyruvate dioxygenase) and inhibit the conversion of 4-hydroxyphenylpyruvate to homogentisate [20]. We, therefore, assayed NTBC treatment for disruption of pyomelanin production in *P. aeruginosa*. The two pyomelanin-producing strains (*hmgA::tn* and PA1111) were grown overnight with increasing amounts of NTBC. Following overnight growth, inhibition of pyomelanin production was determined visually (Fig. 2a). NTBC (300 μM) inhibited pyomelanin production in *hmgA::tn,* while PA1111 required higher concentrations of NTBC to inhibit pyomelanin production (900 μM). Sequencing of *hpd*_PA1111_ revealed two silent mutations upon comparison with *hpd*_PAO1_ (PA0865), demonstrating that mutations within Hpd were not responsible for the residual PA1111 pyomelanin production in the presence of 300 μM NTBC. To ensure that NTBC did not alter the growth of *P. aeruginosa,* we measured the optical densities of cultures grown in the presence and absence of NTBC. The laboratory strains and PA1111 grew at the same rate in the presence or absence of 300 μM NTBC (Fig. 2b), indicating that the reduction in pigmentation was not due to altered growth rates.

### NTBC Treatment of Pyomelanogenic Strains does not Alter Aminoglycoside MICs

It has been reported that melanin has the ability to non-specifically bind a number of diverse compounds. Isotherm analysis indicated that gentamycin had a high level of binding to synthetic melanin through a series of diverse interactions [28]. Melanin–tobramycin interactions have resulted in a decrease of antibiotic activity of 80 % under certain conditions [29]. Aminoglycosides are positively charged at physiological pH, which may contribute to the interactions with negatively charged melanin [6]. Furthermore, a significant correlation was seen between pyomelanin production in *Stenotrophomonas maltophilia* and resistance to specific antibiotics [14]. We therefore assayed both pyomelanin producing and non-producing strains (with and without NTBC treatment) to determine the minimal inhibitory concentrations (MICs) of aminoglycosides.

Minimal inhibitory concentrations were determined by twofold serial microtiter broth dilution [24]. Our results indicated that, under these conditions, the pyomelanin producing strains (*hmgA::tn* and PA1111) did not show significantly higher aminoglycoside MICs than the non-pyomelanin producing strains (PAO1 and *hpd::tn*, Table 1). While treatment of the pyomelanin producing strains with NTBC did inhibit pyomelanin production, the MICs remained unchanged. These data indicated that neither pyomelanin production nor NTBC treatment affect the aminoglycoside MICs for *P. aeruginosa*. This is in agreement with an earlier study wherein MICs were unaltered by pyomelanin production [3], and provides further clarity to the discussion within the literature regarding pyomelanin production and antibiotic resistance. Early studies of pyomelanin production reported that pyomelanogenic *P. aeruginosa* isolates were more sensitive to antibiotics when compared to non-pyomelanogenic strains [30]. In contrast, when *Staphylococcus aureus* was incubated in supernatant from pyomelanogenic *P. aeruginosa*, the MIC values remained unchanged [31]. When considering the results of these studies, it is critical to consider the sources of the melanin; the isotherm analysis was conducted with eumelanin (or synthetic melanin) generated from 3,4-dihydroxyphenylalanine (DOPA), not pyomelanin generated from homogentisate [6]. It is possible that the discrepancy between our results and the isotherm studies is due to the differences in melanin structures (G. Moran, personal communication). While the *S. maltophilia* studies did correlate pyomelanin production with increased resistance to some β-lactam antibiotics and fluoroquinolones, resistance was not detected to either gentamycin or trimethoprim/sulfamethoxazole [14]. Importantly, a direct causal relationship was not tested, and the authors acknowledged that these phenotypes could have resulted from independent mutations.

**Table 1:** Aminoglycoside MICs (μg/ml) of laboratory and clinical isolates of *P. aeruginosa*

	PAO1		*hmgA::tn*		*hpd::tn*		PA1111	
	− NTBC	+ NTBC	−NTBC	+NTBC	−NTBC	+NTBC	−NTBC	+NTBC
Gentamycin	1	0.5	2	2	1	1	0.5	0.5
Kanamycin	16	8	32	32	32	32	16	16
Tobramycin	0.5	0.5	0.5	0.5	0.25	0.25	0.5	0.5

Three independent colonies were tested in triplicate for each strain

### NTBC Treatment of Pyomelanin-Producing *P. aeruginosa* Increases Sensitivity to Oxidative Stress

The antioxidant properties of pyomelanin are proposed to contribute to the increased persistence and virulence of pyomelanogenic bacteria in infection models [3, 11, 12]. Since pyomelanogenic strains of *Burkholderia cepacia* and *P. aeruginosa* have increased resistance to hydrogen peroxide, we examined if NTBC treatment increased sensitivity of pyomelanogenic strains of *P. aeruginosa* to oxidative stress.

**Fig. 3:**
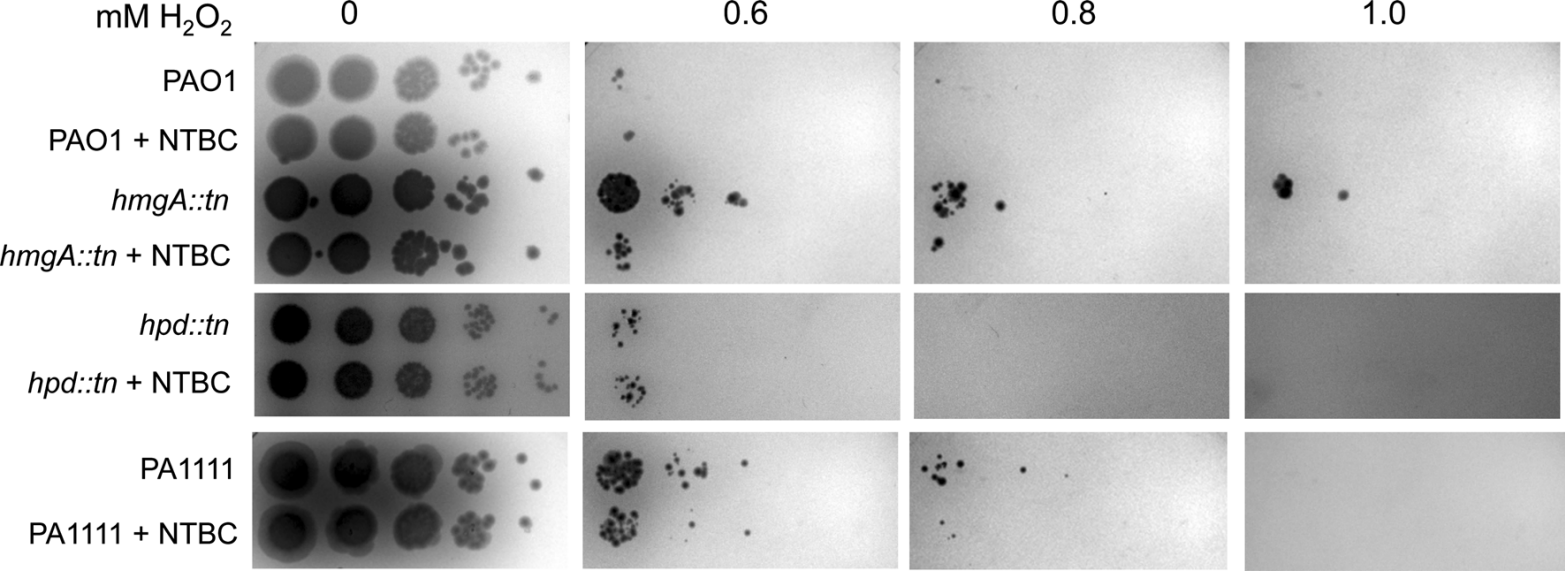
NTBC treatment increases H_2_O_2_ sensitivity in pyomelanin producing strains. 10-fold serial dilutions of the indicated strains were spotted onto LB plates containing the indicated concentrations of hydrogen peroxide

The H_2_O_2_ spot plates showed that both pyomelanogenic strains (*hmgA::tn* and PA1111) have increased resistance to hydrogen peroxide relative to the non-pyomelanogenic strains (PAO1 and *hpd::tn*) (Fig. 3). Importantly, NTBC treatment of pyomelanogenic strains resulted in increased sensitivity to 0.6 mM H_2_O_2_. This illustrates the potential use of NTBC as an anti-virulence factor. The change in sensitivity to H_2_O_2_ was smaller for PA1111 than *hmgA::tn*, and resulted in an approximately 24 % reduction in number of PA1111 colony forming units (based on 4 independent experiments). It is likely that the residual pyomelanin produced in PA1111 at 300 μM NTBC provides a small level of protection against oxidative stress compared to *hmgA::tn*. As expected, NTBC treatment of either wild-type PAO1 or *hpd::tn* did not affect sensitivity to H_2_O_2_.

In this report, we determined that the pyomelanin production in a strain of *P. aeruginosa* PA1111 isolated from an acute infection was likely due to the loss of HmgA activity resulting from a chromosomal deletion [25]. This phenotype has previously been reported for CF isolates and has been shown to decrease clearance/increase persistence in mouse models of chronic infection, suggesting that the development of pyomelanin production may confer an adaptive advantage [3, 10]. Given the antioxidant properties of pyomelanin, it is likely that pigment production would provide protection from oxidative stress in both chronic and acute infections.

This study has shown that NTBC treatment inhibited pyomelanin production by *P. aeruginosa*, and in doing so increased the sensitivity of both laboratory and clinical isolates to oxidative stress, as is found in the respiratory burst from macrophages and monocytes. This suggests that NTBC, as an already FDA-approved compound, has potential as an anti-virulence factor that could be used in combination with existing antibiotics. Pyomelanin is made by a wide variety of organisms, and has been reported in both chronic and acute infections. Given the number of organisms that produce pyomelanin, its functions in iron acquisition and as an antioxidant, and the presence of pyomelanin in both acute and chronic infections, there are a high number of potential applications of NTBC as an anti-virulence factor.

## Electronic supplementary material

Below is the link to the electronic supplementary material.

Supplementary material 1 (PDF 611 kb)
