# Supplementary material for: NTBC Treatment of the Pyomelanogenic Pseudomonas aeruginosa Clinical Isolate PA1111 Inhibits Pigment Production and Increases Sensitivity to Oxidative Stress
Source: Curr Microbiol. 2014 May 7;69(3):343–8. doi: 10.1007/s00284-014-0593-9 (PMC4113677; doi:10.1007/s00284-014-0593-9)
Supplement: Supplementary file 1 — Supplementary material 1 (PDF 611 kb) [file 284_2014_593_MOESM1_ESM.pdf]

Supplementary Materials and Methods

HmgA overexpression. *hmgA* (PA2009) was amplified from the PAO1 genome using primers hmgAfor (5' GGTCTAGAATGAACCTCGACTCCACTGC 3') and hmgArev (5' CCGAGCTCTTATCTCCGTTGCGGGTTG 3') and cloned into the XbaI and SacI sites of pJN105 [1]. The PCR product was sequenced to ensure no mutations were introduced. The resulting plasmids were transformed into *P. aeruginosa* strains. Following confirmation of transformation, HmgA expression was induced with arabinose during overnight growth in LB supplemented with gentamycin.

RT-PCR. Strains were grown overnight until pigment production occurred in pyomelanin producers. RNA was isolated from $5x10^8$ cells using RNeasy Mini Kit (Qiagen) and digested with DNaseI (Promega). RT-PCR was performed using OneStep RT-PCR Kit (Qiagen) with equal concentrations of RNA for all strains tested. Primers were designed to amplify 202 base pairs at the 5' end of *hpd*, beginning with the start codon. The following primers were used: hpD-RT-F (5' ATGAACGCCGTGCCAAGATCG 3') and hpD-RT-R (5' CGTTGAGCACGATGTTGATATC 3'). Primers used for the amplification of 200 base pairs of 16S rRNA are as follows: 16S-RT-PCR-F (5' GACTCCTACGGGAGGCAGC 3') and 16S-RT-PCR-R (5' GTATTACCGCGGCTGCTGGC 3'). Relative amounts of RT-PCR products were estimated using ImageJ software.

Southern Hybridization. Chromosomal DNA was isolated from laboratory and clinical isolates of *Pseudomonas aeruginosa*, digested with SalI, electrophoresed, and transferred to positively charged nylon membranes by a downward capillary transfer method [2]. A digoxigenin-labeled probe was generated through amplification of *hmgA* by PCR and the incorporation of digoxigenin-UTP by random priming as recommended by the manufacturer (Roche). Southern hybridizations were performed as previously described [3].

References

1. Newman J and Fuqua C (1999) Broad-host-range expression vectors that carry the L-arabinose-inducible *Escherichia coli* araBAD promoter and the araC regulator. Gene, 227:197-203.
2. Sambrook J and Russell D (2001) Molecular cloning: A laboratory manual, 3rd ed. 3rd Cold Spring Harbor, N.Y.
3. Thomas N, Pawson C, and Jarrell K (2001) Insertional inactivation of the *flaH* gene of the archaeon *Methanococcus voltae* resuls in non-flagellated cells. J Bacteriol, 183:7154-64.

Online resource 1.

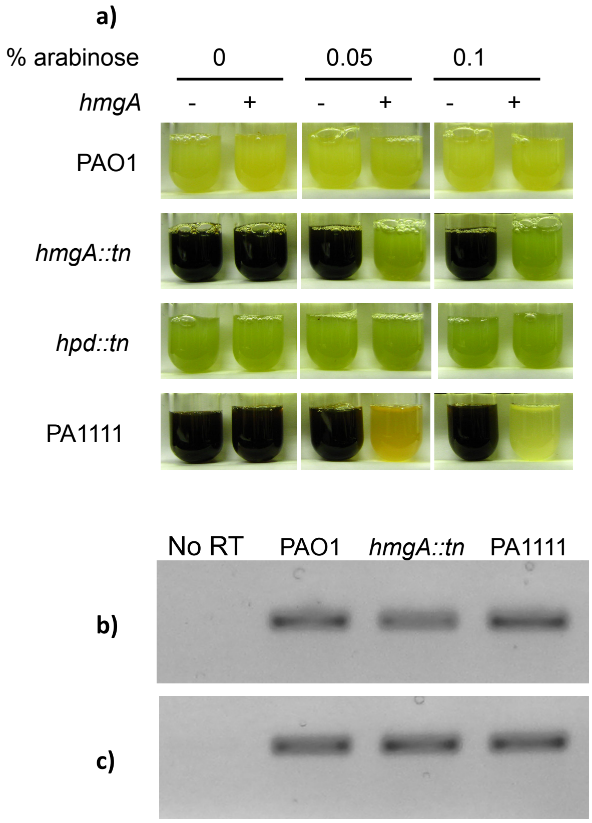

Online resource 1 HmgA expression alleviates pyomelanin production in lab and clinical isolates in a dose dependent manner. **a)** The indicated *P. aeruginosa* strains containing either *hmgA*-pJN105 (+) or pJN105 (-) were incubated overnight in LB + gentamycin (50 µg/ml) with the indicated concentrations of arabinose. **b)** RT-PCR amplification of *hpd* transcript in PAO1, *hmgA::tn*, and PA1111. **c)** RT-PCR amplification of 16S rRNA from PAO1, *hmgA::tn* and PA1111
